# A Beam-Specific Optimization Target Volume for Stereotactic Proton Pencil Beam Scanning Therapy for Locally Advanced Pancreatic Cancer

**DOI:** 10.1016/j.adro.2021.100757

**Published:** 2021-07-29

**Authors:** Dong Han, Hamed Hooshangnejad, Chin-Cheng Chen, Kai Ding

**Affiliations:** a Departments of Radiation Oncology and Molecular Radiation Sciences, Johns Hopkins School of Medicine, Baltimore, Maryland; b Departments of Biomedical Engineering, Johns Hopkins School of Medicine, Baltimore, Maryland; c Departments of Radiation Oncology and Molecular Radiation Sciences, Johns Hopkins Proton Therapy Center, Washington, District of Columbia; d Maryland Proton Treatment Center, Departments of Radiation Oncology; University of Maryland School of Medicine, Baltimore, Maryland

## Abstract

**Purpose:**

We investigate two margin-based schemes for optimization target volumes (OTV), both isotropic expansion (2 mm) and beam-specific OTV, to account for uncertainties due to the setup errors and range uncertainties in pancreatic stereotactic pencil beam scanning (PBS) proton therapy. Also, as 2-mm being one of the extreme sizes of margin, we also study whether the plan quality of 2-mm uniform expansion could be comparable to other plan schemes.

**Methods and Materials:**

We developed 2 schemes for OTV: (1) a uniform expansion of 2 mm (OTV_2mm_) for setup uncertainty and (2) a water equivalent thickness–based, beam-specific expansion (OTV_WET_) on beam direction and 2 mm expansion laterally. Six LAPC patients were planned with a prescribed dose of 33 Gy (RBE) in 5 fractions. Robustness optimization (RO) plans on gross tumor volumes, with setup uncertainties of 2 mm and range uncertainties of 3.5%, were implemented as a benchmark.

**Results:**

All 3 optimization schemes achieved decent target coverage with no significant difference. The OTV_2mm_ plans show superior organ at risk (OAR) sparing, especially for proximal duodenum. However, OTV_2mm_ plans demonstrate severe susceptibility to range and setup uncertainties with a passing rate of 19% of the plans meeting the goal of 95% volume covered by the prescribed dose. The proposed dose spread function analysis shows no significant difference.

**Conclusions:**

The use of OTV_WET_ mimics a union volume for all scenarios in robust optimization but saves optimization time noticeably. The beam-specific margin can be attractive to online adaptive stereotactic body proton therapy owing to the efficiency of the plan optimization.

## Introduction

Pancreatic adenocarcinoma is one of the most lethal cancers and has a 5-year survival rate of less than 10%.^1^ To improve the overall survival, there is increasing interest in exploring the hypofractionated regime of radiation therapy in treating local advanced pancreatic cancer. Stereotactic body radiation therapy (SBRT) is one of the techniques allowing delivery of higher dose to target and steeper dose falloff to normal tissues in 3 to 5 fractions, thereby maximizing the therapeutic ratio. The alternative treatment options are to deliver an ablative dose in 15 to 28 fractions^2^ for better local control.^2^

Proton therapy has the clinical advantage of depositing the entire prescription dose to the target and yielding no exit dose. Therefore, proton therapy can potentially reduce the dose to normal tissues, resulting in ameliorated local control and decreased toxicities. The study by Thompson et al^3^ shows that compared with conventional photon therapy, proton plans could significantly decrease the low and intermediate dose to critical organs (ie, duodenum, stomach and liver, etc), while maintaining the dose levels to target. Craneet al^2^ show that proton therapy can reduce exposure to normal tissue compared with intensity-modulated radiation therapy with 10 -15 mm margin size from GTV to PTV for pancreatic head cancer. Bouchard et al^4^ confirm this finding and claim it is feasible to boost the therapeutic ratio. Recently, Jethwa et al^5^ implemented a dosimetric analysis between intensity modulated proton therapy (IMPT) plans and volumetric modulated arc therapy plans for localized intact pancreatic cancer, showing IMPT offers superiority to intensity-modulated radiation therapy in reduction of dose to OARs.

Combining the techniques of SBRT with proton therapy as an emerging novel technique is believed to further boost the gain of local control and minimize toxicity.^6^ Sio et al^7^ provided a systematic quantitative comparison between photon stereotactic body radiation therapy (SBRT) and proton stereotactic body proton therapy (SBPT), and the results suggest comparable organs at risk (OAR) sparing in the high-dose region and improved dosimetric sparing for low- and medium-dose regions.

To accurately deliver the prescribed proton dose to the target, one must incorporate setup and range uncertainties. Owing to the inherent uncertainty of conversion of computed tomography (CT) image to stopping power, a range uncertainty of 3.5% is often considered in treatment planning.^8^, ^9^, ^10^ For instance, for margin-based treatment planning, a target volume used for optimization target volumes (OTV) iscreated from expansion from clinical target volume or gross tumor volumes (GTVs) . Sio et al^7^ compared the plan quality of 3 mm, 5 mm, and 7 mm expansions for SBPT in pancreatic cancer. Thompson et al^3^ used the expansion of 5 mm as photon therapy to produce OTV for proton pencil beam scanning (PBS). A beam-specific margins tehcnique is proposed to account for proton beam range uncertainties explicitly for by Park et al.^11^ They tested this technique on prostate cases to demonstrate its superiority to the geometric margin used by Thompson et al^3^ and Sio et al^7^ for target coverage. However, the efficacy and robustness of this method on the abdominal case remain to be investigated.

Other than designing margins explicitly to account for uncertainties in proton therapy, a novel way of treatment planning that incorporates the uncertainty into the process of optimization was recently introduced,^12^ namely robustness optimization (RO). It aims to achieve a robust dose distribution that is insensitive to setup and range uncertainties. The input parameters are determined based on the scenarios of estimated setup and range margins. For example, during the optimization process, to account for range uncertainties, the optimizer automatically expands the margin implicitly without specifying the OTV.^5^^,^^9^ If an OAR is adjacent to the target, instead of placing the margins directly, the optimizer will place dose falloff to shape the dose distribution.^9^ Although robust optimization outperforms margin-based methods in the robustness of target coverage,^13^ a large number of error scenarios impose a huge computation burden on clinical implementation of robust optimizations.

The amount of setup-uncertainty margins used in margin-based planning or robustness optimization planning are often derived from imaging guidance (ie, stereoscopic, kilovoltage, 2-dimensional x-ray imaging).^5^ With the help of 3-dimensional imaging guidance and surrogate fiducials implanted close to the lesion, the setup uncertainties can be further reduced. It is yet known how the reduction of setup margin can benefit the robustness optimization process of proton treatment planning for abdominal targets. To battle with the duodenum toxicity, a novel, absorbable iodinated polyethylene glycol–based hydrogel for tissue marking and spacing was studied to further increase the separation between panceas and duodenum.^14^^,^^15^ The feasibility of incorporating this spacer into proton planning has not systematically investigated. As a first step, if the mere uncertaines other than range uncertainty are studied, it remains unclear whether the spacer can behave a “buffer” to help to reduce the duodenum toxicity, as in photon therapy.

We also investigated 2 margin-based optimization schemes, isotropic expansion and beam-specific OTV, to account for uncertainties associated with setup errors and range uncertainties in pancreatic stereotactic PBS proton therapy. The size of the expansion is inferred from the tolerance of the 3-dimensional cone-beam CT imaging guidance device used in our center. The robustness, target dose coverage, and OAR sparing of the plans generated by the 2 margin-based optimization schemes are quantitatively compared with plans using robustness optimization.

## Methods and Materials

Six patients with clinically diagnosed, localized, advanced pancreatic adenocarcinoma who underwent SBRT active breath control technique were chosen in this planning study. All patients were positioned in supine with arms above the head under breath hold during the simulation.

The GTV, OAR, i.e. duodenum, small bowel, etc were contoured by physicians. GTV volumes ranged from 7.6 mL to 82.2 mL. The OARs, including duodenum, small intestine, stomach, kidneys, and spinal cord, were delineated. The CT images and structures were imported into Raystation 9A (RaySearch Laboratories AB, Stockholm, Sweden) for PBS proton treatment planning.

All patients were prescribed with a total dose of 33 Gy (RBE) in a treatment course of 5 fractions. The nominal goal of dose coverage of the GTVs should be 98% of volume receiving at least 100% of the prescribed dose (D98). More details can be found below for each optimization scheme. The OAR constraints include the volume of receiving 100% prescribed dose V_33Gy(RBE)_ < 1 cm^3^; V_20 Gy__(RBE)_ < 20 cm^3^ and V_15 Gy (RBE)_ < 15 cm^3^ in compliance with our institutional guidelines.

### Beam configuration

The plans were designed to be delivered via PBS using the Hitachi Probeat-CR system equipped with volumetric imaging guidance of the cone-beam computed tomography system. Two posterior oblique fields at gantry of 210 and 150 degrees were used in the treatment planning for all patients except one, who was planned with a posterior (180) and posterior oblique beam (150) to reduce dose to the right kidney. The spot scanning pattern is set hexagonal. The spot size is decided by the optimization algorithm. The use of range shifter is determined on a patient-specific basis.

### OTV definitions

Two OTV schemes were investigated. The first scheme is the OTV_2mm_, which was designed in a fashion of geometric uniform 2-mm expansion around the GTV. With the help of cone-beam computed tomography and implanted fiducial markers, a tolerance of 2 mm was then used in the treatment planning process for the setup uncertainty. The second scheme is a patient-specific scheme (OTV_WET_), proposed by Park et al^11^ and based on water-equivalent thicknesses (WETs). Along the beam direction, distal and proximal WETs were determined based on the stoichiometric calibration method and converted to geometric margins by multiplying 3.5% added to GTV as the OTV_WET_. On the beam's eye-view directions, an expansion of 2 mm was used to account for lateral setup uncertainty.

### Plan optimization

The OTV_2mm_ and OTV_WET_ were used for the target dose coverages in the plan optimization. The homogeneous dose objectives (minimum, maximum, and uniform doses) were applied to the whole OTV_2mm,_ and 2 beam-specific OTV_WET_ with equal dose weight. The OTV-optimized plans were compared with the robustness optimization (RO) method, which incorporates the setup and range uncertainties. In this study, a setup uncertainty of 2 mm and a range uncertainty of 3.5% on GTV were used in the RO optimization process. A total of 42 scenarios have to be computed to account for all possible uncertainties, including isocenter with no shifts, and shifted toward patient's anterior, posterior, right, left inferior directions, and diagonal directions (14 scenarios), each of which is with 3 scenarios of −3.5%, 0, +3.5% scaling of WET. The single-field optimization was used for all plans to have the uniform dose distribution in target volumes from each field.

### Plan evaluation

The target (GTV) dose coverage of the target volume (GTV) and the dose sparing of OARs were compared among 3 schemes for 6 patients. A Bonferroni-corrected *t* test was used to evaluate the statistical significance.

There is an agreement that plan robustness as a plan quality metric should be included.^16^ Although there is no consensus on what exact scenarios should be included in the evaluation for robustness evaluation,^16^ a separate exam of setup and range uncertainties were used. To evaluate the dose coverage of target under the various conditions of uncertainties, simultaneously 14 setup errors (in both parallel and diagonal directions) and 3 range uncertainties (including a nominal plan with no range uncertainty) with 42 totality of scenarios are examined. All plans aim to achieve at least 95% of the volume of the GTV receiving 100% of the prescribed dose.

The plan robustness could also be evaluated using the dose-volume histogram (DVH) deviation, “bandwidth,” due to the perturbations of isocenter shift and range uncertainties for target and duodenum. A separate independent of 6 setup errors and 3.5% for an under and over range were analyzed. Of all the 8 scenarios, the bandwidth of DVH is defined as the standard deviation at specified dose parameters.

(1) $$W_{d}=\frac{2\sqrt{\frac{\sum_{i=1}^{N}{\left(D_{i}-D_{no\min al}\right)}^{2}}{N-1}}}{D_{no\min al}}\%$$

**Figure 1:**
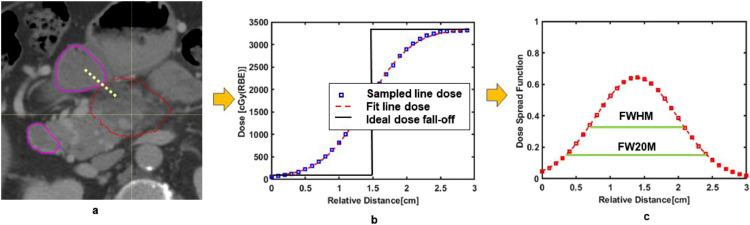
(a) An isodose line of 100% (solid red line) is next to the duodenum (solid purple line). (b) A sampled line dose profile according to the yellow dashed is shown. The relative distance is calculated from either end of sampled dose line. (c) The dose spread profile is shown with full-with-half-maximum (FWHM), full-width-20%-maximum (FW20M).

Because one of the most main critical structures duodenum, abuts tumor target, a quantity, termed as dose spread function, was proposed to describe dose falloff in the abutment region. In Fig. 1a, the spatial relationship between a typical 100% iso-dose line and duodenum is shown. A line dose profile can be sampled and shown in Fig. 1b. The dose profile can be obtained by fitting the sampled line dose. By differentiating the fitting line dose, the dose spread function (DSF) can be analytically obtained. Mathematically, this can be expressed as follows:

(2) $$DSF=\frac{d}{dr}\left(LD\right)$$

Here *r* is the distance from either end of the dose line. *LD* refers to the line dose profile. Via DSF, the steepness of the dose falloff gradient, can be described quantitatively. For example, on Figure 1c, the FWHM is referring to the width needed to reduce 100% dose to 50%, whereas FW20M presents the width for the dose to drop from 100% to 20%. To show the practical use of DSF, the line dose, representing dose dropping from 100%, is chosen to cross the abutment region between 100% isodose to duodenum structure. The dose distribution, DVH and line dose profile were exported from Raystation TPS and analyzed by using in-house MATLAB tools.

## Results

**Figure 2:**
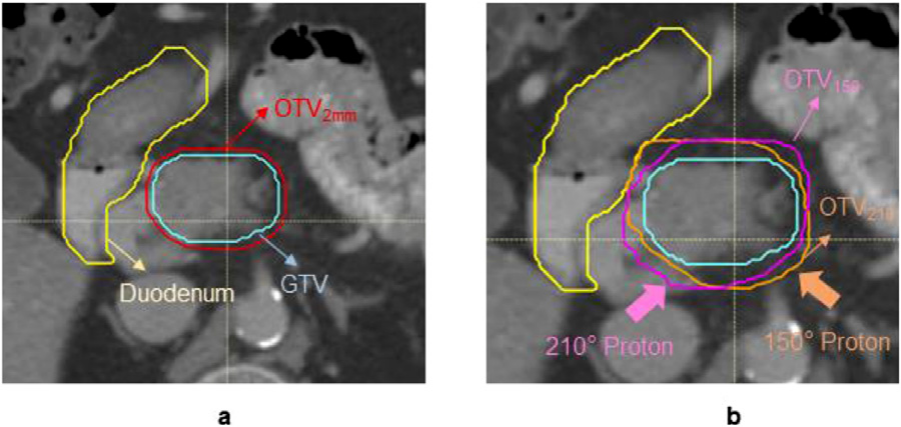
The gross target volume (GTV) in the red solid is used to derive margins for (a) OTV_2mm_ (optimization target volume) and (b) OTV_WET_. The expansions for OTV_WET_ are beam specific. The overlaps between the anatomical structure and OTV are excluded. The pink shall be OTV_210_ and orange is OTV_150_. *Abbreviation:* GTV= gross target volume; OTV = optimization target volumes.

The derived OTV_WET_ has a more substantial expansion: >2 mm from GTV due to targets being deep-seated in general. It is estimated that the geometric distance from the beam entrance to GTV distal is about 16 to 19 cm. Not like uniform expansion, OTV_WET_ has less conformality to target due to the expansion for range uncertainties. This can be observed from one of the patients shown in Figure 2. The more expansion in the beam direction, the more degradation of dose conformality can be seen in Figure E1 a of the Supplementary Materials.

**Figure 3:**
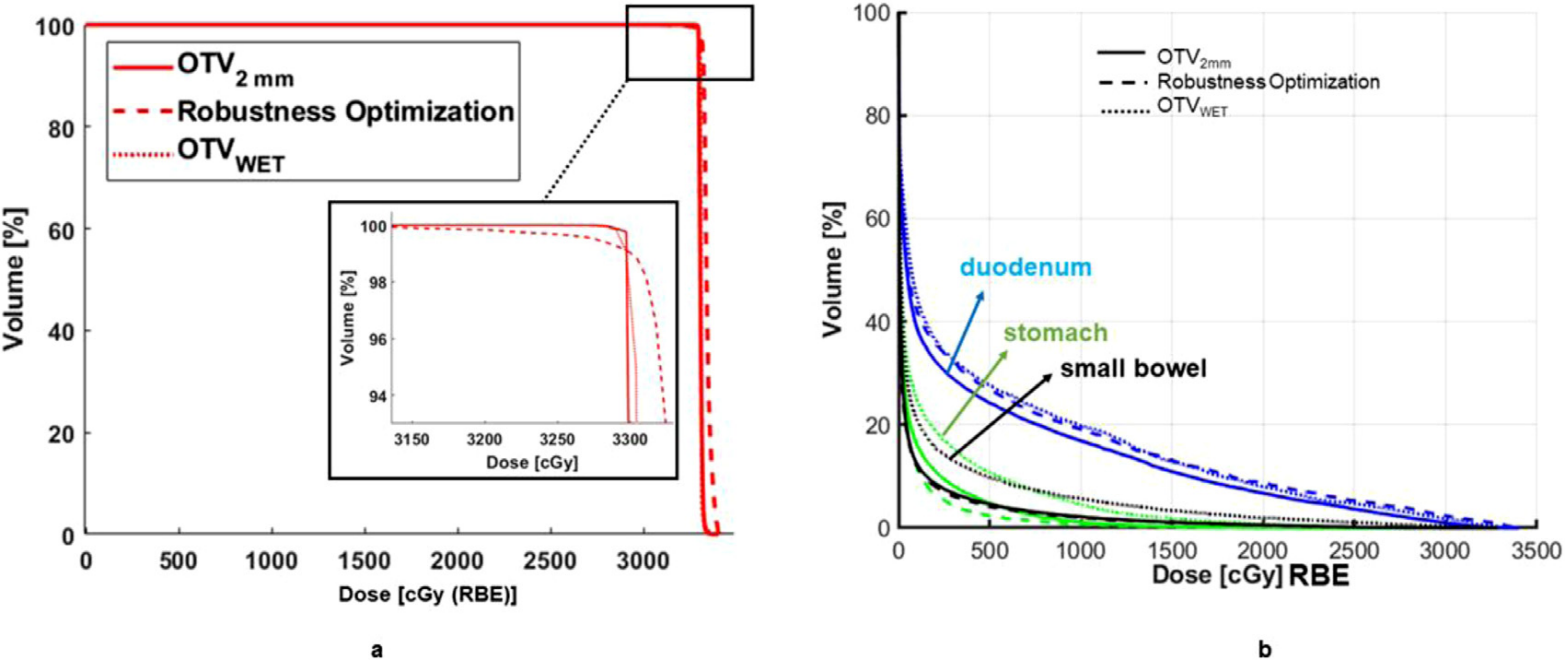
(a) A typical dose-volume histogram of gross target volume for respective planning of 3 schemes. Similar coverage can be observed among all 3 plans, and the goal of target coverage is met. (b) The dose-volume histogram for the organs at risk of the duodenum, stomach, and small bowel are shown for plans of OTV_2mm_ (optimization target volume), OTV_WET_, and robustness optimization. *Abbreviation:* OTV = optimization target volumes.

Figure 3 a exemplifies the DVH similarities for target coverage among 3 plans for one of the patients. Table E1 in the Supplementary Materials summarizes the results of the planning comparison. DVHs of the GTV show that for D98, D_max_ and D_mean_, these 3 plans have very similar coverage. As expected, no statistically significant differences among the plans were found for GTVs, except D98 for RO and OTV_WET_ plans. A slightly larger D_max_ can be found for the plan using robust optimization (RO plan).

Figure 3b shows an example of dose sparing for critical OARs achieved by 3 plans. Due to a smaller margin than OTV_WET_, the OTV_2mm_ yields the best dose avoidance for duodenum in the high, medium, and low dose regions. In addition to the setup uncertainties of 2 mm, the RO plan also considers the range uncertainties explicitly, leading to the performance of dose sparing similar to the plan of OTV_WET_. However, in this case, the plan of OTV_WET_ is the worst of sparing small bowel compared with the other 2. According to Table E1 in the Supplementary Materials, OTV_2mm_ plans show statistically significant in dose sparing of the duodenum and small bowel over OTV_WET_ due to the smaller margin in OTV_2mm_. The plans of OTV_2mm_ also outperform the RO plans in high dose region sparing for small bowel and stomach.

**Figure 4:**
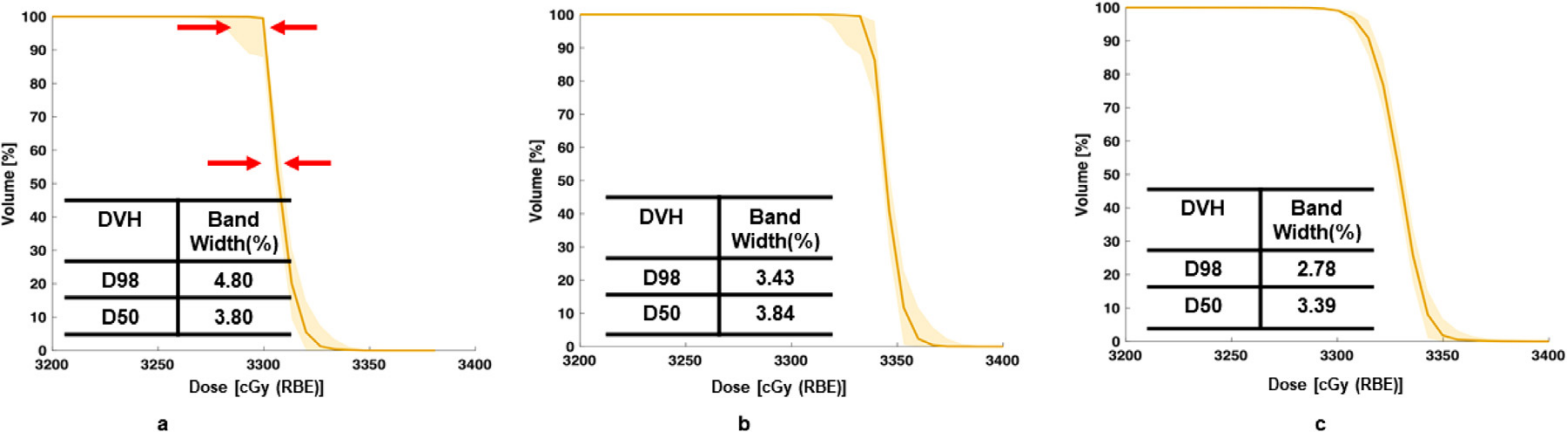
Dose-volume histograms (DVH) for target coverage perturbed by simulated scenarios of setup errors and range uncertainties for (a) OTV_2mm_ (optimization target volume); (b) OTV_WET_; and (c) RO plans. *Abbreviation:* OTV = optimization target volumes.

Among all the plans, the response of the nominal plans to perturbations may be plan scheme specific. For example, for target coverage in Figure 4, the compactness or bandwidth of D98 and D50 on DVH shows the least for RO plans. It demonstrates that the RO plans are more forgivable to the setup and range uncertainties as expected. The bandwidths of OTV_2mm_ and OTV_WET_ are similar, mainly, for D50, the widths are 3.80% and 3.84% for OTV_2mm_ and OTV_WET_, respectively. In other words, the worst resultant target dose could be under- or overdose around 4%. The bandwidths at D98 for OTV_2mm_ and RO are 4.8% and 2.8%, respectively. For this case, the loss of the plan quality due to the perturbation could be up to around 5% for OTV_2mm_, while for RO plan, such loss could be around 3%. According to our plan objectives, the goal of the target coverage could be maintained.

**Figure 5:**
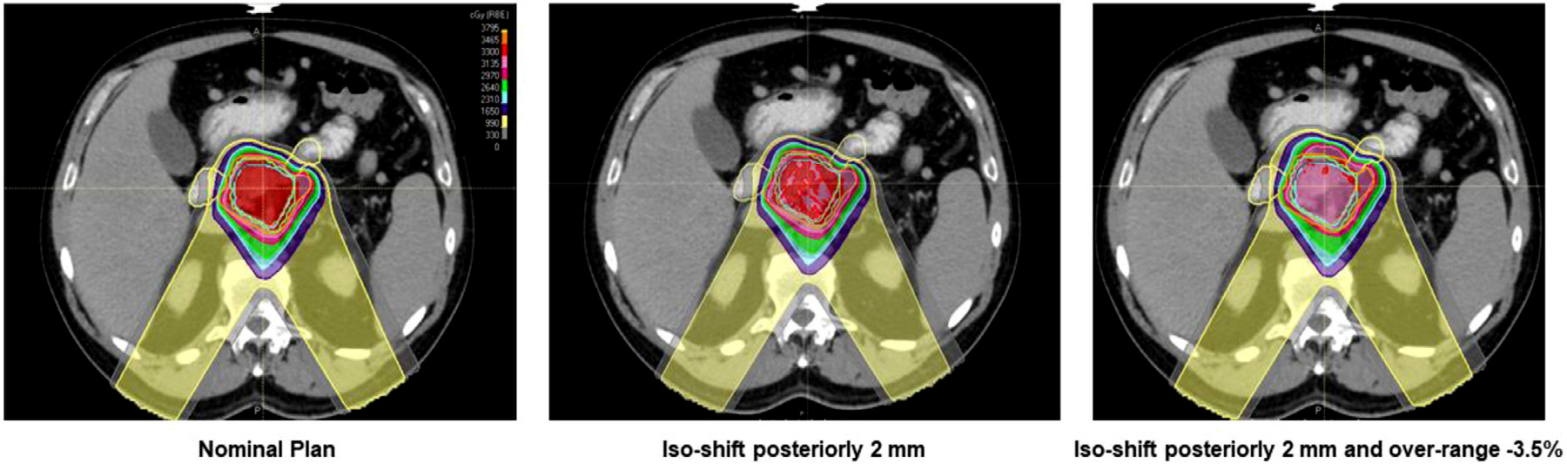
Target dose of OTV_2mm_ (optimization target volume) from one of the patients shows vulnerability to simulated setup and range uncertainties. For one of the cases above, the D95 coverage of gross target volume has been degraded to <5% if simultaneous setup errors and range uncertainties exist, and 2 mm expansion fails to compensate the total errors.

The average passing rate of target coverage for all patient with the goal of 95% target covered by 100% prescribed dose is only 19% in OTV_2mm_ compared to 100% for OTV_WET_ and RO. This result shows that OTV_2mm_ is not robust enough to offset the perturbation to the dose coverage. One example can be found in Figure 5, where an intentional isocenter shift posteriorly 2 mm and overranged by 3.5% are introduced. In this case, the target dose coverage (D95) can dramatically drop to 70%, leading to huge plan quality degradation.

**Figure 6:**
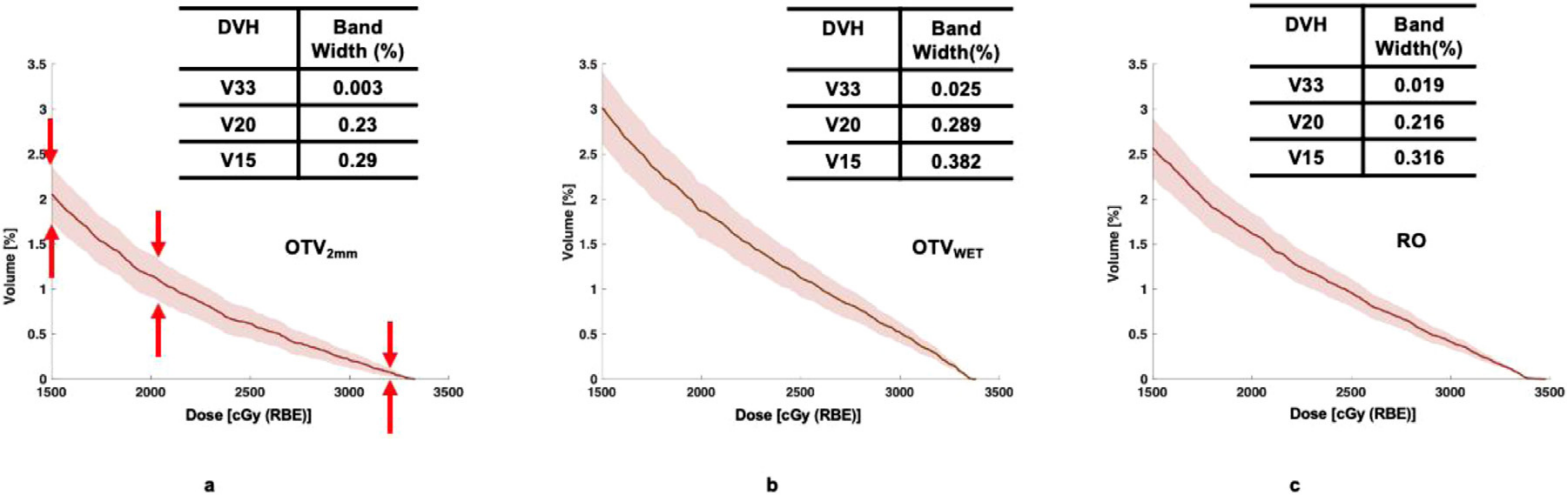
Dose volume histogram (DVH) of duodenum perturbed by simulated scenarios of setup and range uncertainties for (a) OTV_2mm_ (optimization target volume); (b) OTV_WET_; and (c) RO plans. The bandwidth defined with standard deviation of the nominal dose is referred to in equation 1. Abbreviation: RO = robustness optimization; OTV = optimization target volumes.

The impact of perturbation on OAR (i.e. duodenum) could be revealed in the example case of Figure 6. The OTV_WET_ (b) plan has the most significant bandwidth of medium-dose V_20Gy(RBE)_ among the 3 plans. In the high dose region, the use of OTV_2mm_ (a) has more advantages compared with the other plans in response to perturbation scenarios. Again, this is due to a smaller margin of plans of OTV_2mm_. For the low dose region, 3 plans show similar performance.

Table E2 in the Supplementary Materials summarizes the statistical significance of the resultant impact on the duodenum. For V_20Gy(RBE)_, OTV_2mm_ plan shows the difference from the RO plans. No other significant difference was found among the 3 plans.

The DSF from the RO plan in Figure E2 in the Supplementary Materials shows slightly larger than the other 2, indicating that RO has a more gradual dose falloff region on this particular dose plane. In Table E3 in the Supplementary Materials OTV_2mm_ shows the fastest dose-off among all the patients and plans. For FW20M, the RO plans need about more than 2 mm space to reduce the dose to 20% of the prescribed.

## Discussion

Designing an appropriate margin for treatment plan optimization in proton therapy is quite challenging.^9^^,^^10^^,^^16^ Although accurate in delivery of high dose to target, the proton beam is also sensitive to any variation along the beam path. Such variation could be due to change of anatomy, setup, and range uncertainties.^17^ Thus, image guidance in proton therapy plays a crucial role in clinical to achieve the intended treatment plan dose distribution.

The plan quality of OTV_2mm_ shows it not suitable for clinical use. Our motivation to attempt an aggressive 2 mm margin size in this study is rooted in 3 aspects. First, during the SBRT planning, a higher dose to the tumor while a relatively lower dose to the OARS is highly desired. SBRT is ideally suitable for tumors in parallel organs. The pancreas, however, is in close promity to serially functioning OARs (ie, duodenum, small bowel). The ablative BED dose around 106 Gy with current fractionation scheme to these OARs can produce impairment in the organ function. As such, limiting the OAR dose in LAPC SBPT has become increasingly crucial to the improvements in oncological endpoints.^18^ A more aggressive reduction of margin size is an alternative approach to sparing more OARs dose.

Second, it is worth noting that OTV_2mm_ plan is sensitive to range uncertainties owing to its sufficient margin size to cover the uncertainty along the beam direction. One way to improve the robustness and maintain the low toxicity OTV_2mm_ to duodenum is to place hydrogel not only in the space between duodenum and pancreas, but also along the beam direction by any means. In other words, the function of hydrogel in proton therapy could be viewed as 2-folds: to reduce the toxicity and uncertainties.

Third, the geometric limit of combing fiducial markers and a developed real-time gated floruoscopy at our institution could be within 2 to 3 mm.^18^, ^19^, ^20^ Therefore, as a motion management strategy, OTV_2mm_ as the extreme case of margin design is investigated, whereas this aggressive margin size is not realistic in current proton community.

**Figure 7:**
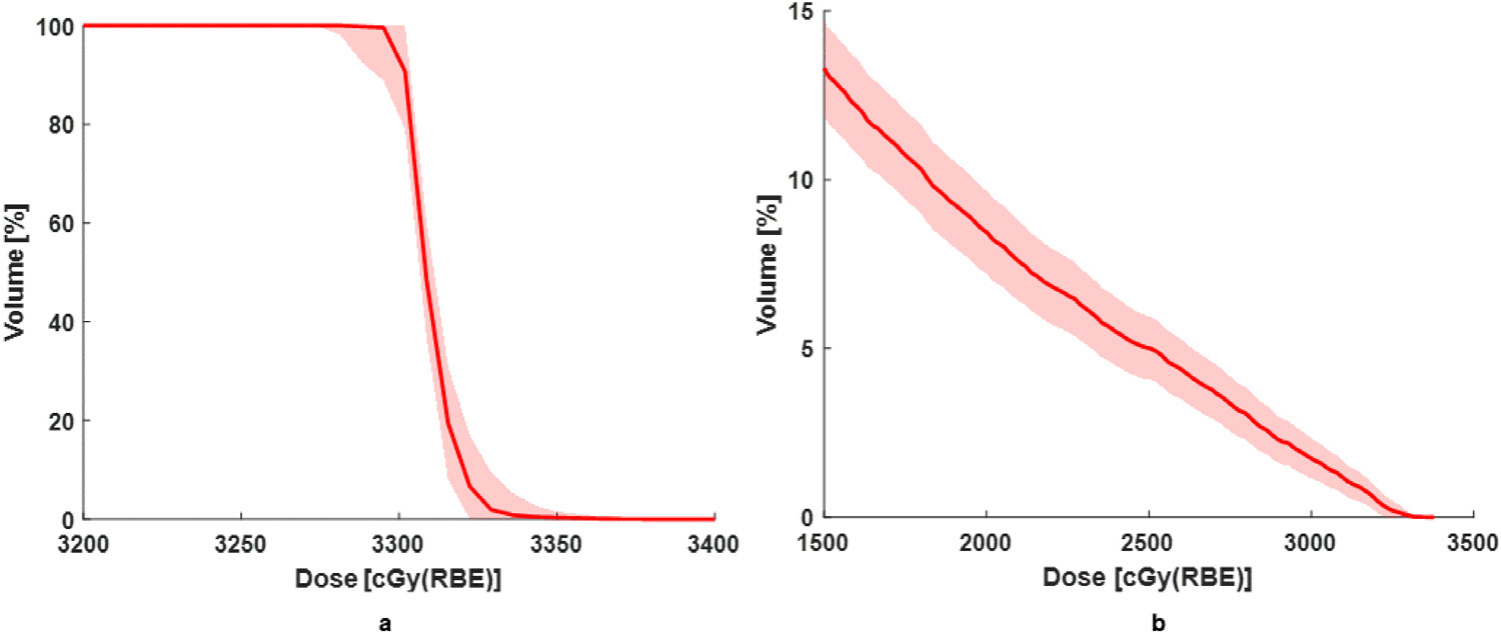
The norminal and perturbed plans for (a) target and (b) duodenum demonstrated for one of the OTV_5mm_ (optimization target volume) plans. *Abbreviation:* OTV = optimization target volumes.

In line with tolerance allowed by the 2-dimensional imaging device, various sizes of margins up to 5 mm or more expansions from clinical target volume or GTV for pancreas proton therapy have been implemented.^3^^,^^5^^,^^7^^,^^21^ As for comparison with OTV_2mm_, we implemented OTV_5mm_ plan study as well. The summary of all OTV_5mm_ plans is updated in Table E1 in Supplementary Materials. One of the patient plans has been shown in Figure 7.

It can be seen that target coverage (D98) is similar to Figure 4, but the overdose of duodenum could be up to 5 times compared with OTV_2mm_. The evaluation of the robustness shows that using a 5-mm margin size is immune to the perturbations of 2-mm setup errors and 3.5% range uncertainties. In this study, it is our expectation that with larger margin size, we are sacrificing OAR sparing for robustness. The robustness evaluation for OTV_5mm_ plans shows resiliency of the plans to the setup error of 2 mm and range uncertainties with passing rate close to 100%.

**Figure 8:**
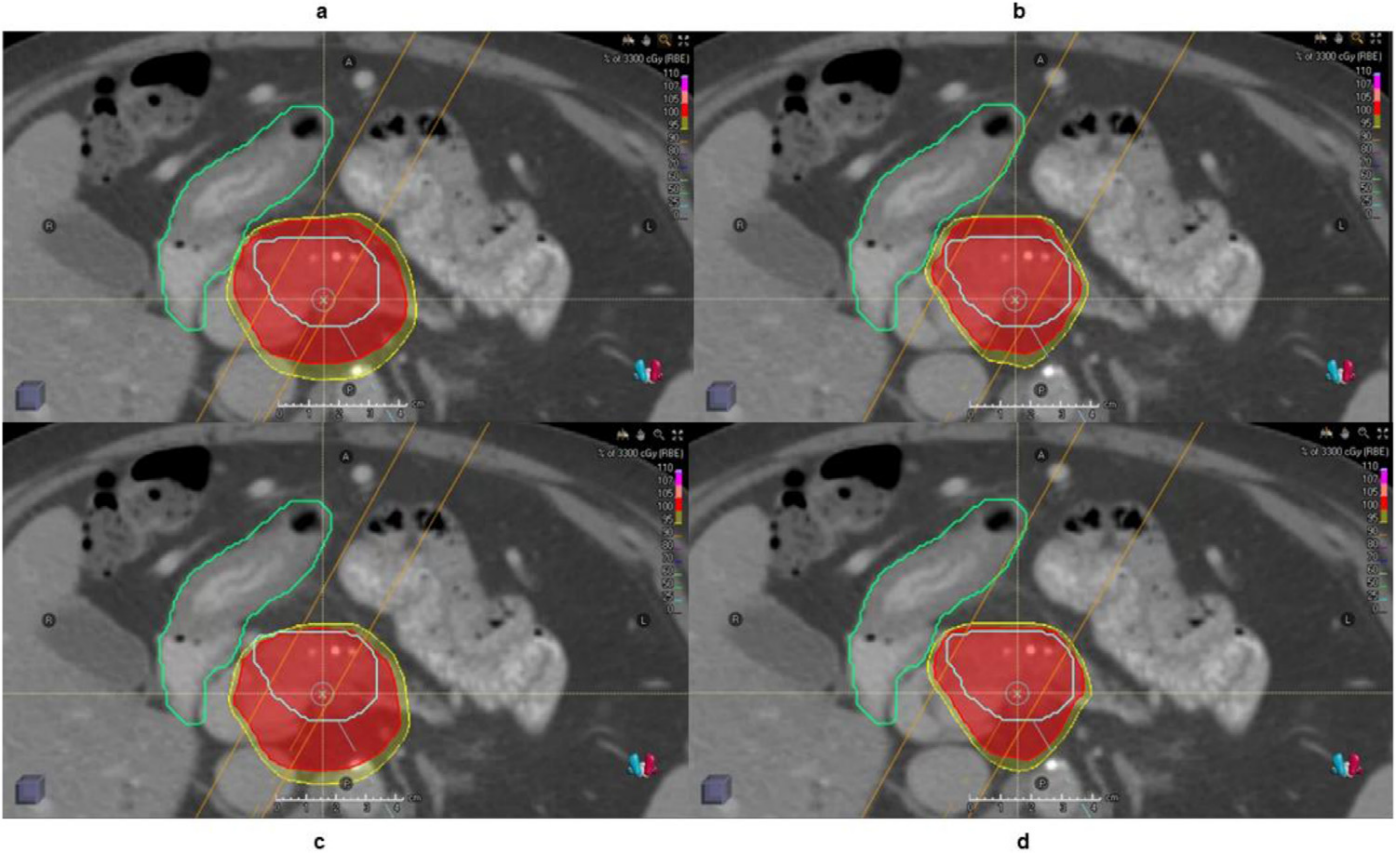
A case of plan falloff comparison between (a) OTV_WET_ (optimization target volume) and (b) robustness optimization along the beam direction shown in orange. The organ at risk (duodenum) is shown in green, and the target is colored with blue. The cloud in red and yellow correspond to 100% and 95% prescription dose, respectively. The undershoot dose for OTV_WET_ and robustness optimization is shown in (c) and (d). *Abbreviation:* OTV = optimization target volumes.

Owing to the combined uncertainties of both patient positioning and proton range, the construction of a beam-specific OTV is more appropriate.^22^ In addition to the margin-based a priori method OTV_WET_, a robustness optimization that considers the range uncertainties and setup error without specifying the margins for optimization was proposed and implemented.^9^^,^^12^ By minimizing the maximum of objective function for scenarios accounting for setup and range uncertanities, robustness optimization could yield plans that are resilient to the perturbation within the specification. Comparing the plans between OTV_WET_ and RO in DSF, it was found that RO plans have less dose gradient in the beam direction. For the case shown herein, the dose falloff is roughly steeper for RO plan than OTV_WET_. As a result, when beams overshoot or undershoot, the dose distribution can be only moderately affected by shifting dose distribution as shown in Figure 8. In other words, RO plan can automatically extend the irradiated region distally along the beam direction to compensate the dosimetric perturbations introduced by range uncertainties. Meanwhile, RO plans can reshape the dose distribution accordingly.^9^ The OTV_WET_ plans predefine the margin with priori knowledge of range uncertainties and shift the dose distribution.^22^ In the Figure 8c, this is demonstrated by a bare coverage of prescribed dose to GTV.

However, one disadvantage of this method is that it requires computation of all the scenarios to determine the maximum of the objective function. In other words, it could be rather time-consuming to minimize or optimize the objective function for a plan. For instances, using Monte Carlo optimization with the running time about 2 hours, while using beam-specfic OTVwet method, the computation time can be decreased to within a half hour. In the pipeline of online adaptive proton therapy, robustness optimization becomes clinically challenging.^23^

Robustness optimization and margin-based plans achieve a similar target dose coverage in D98, D_mean_. In terms of OAR dose sparing of the duodenum, OTV_2mm_ outperforms the other types of plans owing to its smaller expansion. On the other hand, OTV_WET_ plans show inferior to other plans owing to the relatively large expansion. However, the RO plans are robust to setup and range uncertainties without sacrificing the quality of the plans too much. The OTV_2mm_ plans fail most of the scenarios in the robustness evaluation owing to the plans that are extremely sensitive to the perturbations from range uncertainties. The OTV_WET_ plans are insensitive to setup errors and range uncertainties for the beam arrangements used in the pancreatic patients. The dose spread function proposed in the study reveals that RO plans have less gradient of dose falloff at the specified location, indicating that more room between critical structure and 100% isodose line is needed to spare the duodenum in general. The OTV_2mm_ and OTV_WET_ plans have only one scenario to compute, while the RO scheme has 21 scenarios. If the Monte Carlo dose engine was used, it is anticipated that the RO process would take even longer.^24^ In summary, the quality of OTV_WET_ plans falls in the category between OTV_2mm_ and RO; it has similar target dose coverage compared with the other 2 but has the inferior normal tissue sparing compared with OTV_2mm_.

The OTV_WET_ plan explicitly accounts for setup and range uncertainties; it is less susceptible to dosimetric perturbation. As indicated in the results, OTV_WET_ plans have the degradation of quality in robustness evaluation, although the light computation is maintained. In general, the plan quality of OTV_WET_ is closer to RO plans than that of OTV_2mm_ plans.

The proposed quantity DSF can correlate the spatial information of anatomical structure and dose distribution. The edge spread function (ie, dose profile) is used to quantify the dose-response at the edge of the critical structure. At the same time, DSF indicates the rate of dose falloff on the selected image with spatial and directional information. There are other useful tools to quantify the dose falloff behavior,^25^ which may only focus on the distance between the isodose lines, not on the simultaneous effect of the anatomic specific and dosimetric performance. It is noted that recent studies from Rao et al^14^, ^15^, ^26^, ^27^, ^28^, ^29^ showcased a novel biodegradable hydrogel material injected at head-of-duodenum interface that can limit the dose to the duodenum. However, it is necessary to correlate the amount of injected hydrogel with the dosimetric consequence for each patient. By combining the information from DSF in a volumetric format on a patient-specific basis, the injection of the hydrogel can be optimized more accurately. This will remain in our future studies.

In this study, the WET calculation is based on the stochiometric conversion from single-energy CT images, which is believed to introduce 3.5% uncertainties.^8^^,^^30^ To further improve the dose sparing, a possible method is to incorporate dual-energy CT images into this study. It is accepted that the DECT technique can decrease the range uncertainty from 3.5% to 2%.^31^, ^32^, ^33^, ^34^, ^35^, ^36^, ^37^

To the authors’ best knowledge, this study is the first one to compare the 2 types of proton treatment planning techniques for pancreas cancer: margin-based versus robustness optimization. However, this study has several limitations. First, the optimization of beam angle selection is not implemented, as it shows an essential role in the quality of the plans.^22^ Second, the dose falloff region of interest is selected at the abutment of 100% isodose line and duodenum. The choice of this region could be optimized in future investigation.^38^

## Conclusions

We evaluated the plan quality among 3 optimization schemes using uniform expandsion OTV_2mm_, beam-specific OTV_WET_, and robustness optimization on GTV. Although the margin-based method (OTV_2mm_) provides superiority in fast computation and low toxicity, the quality of plans fails to match the ones from robustness optimization in the evaluation of susceptibility to perturbation. A beam-specific margin also remains further investigations for online adaptive stereotactic body proton therapy owing to the efficiency of the plan optimization.

## Appendix Supplementary materials

Image, application 1

Image, application 2

## Footnotes

Supplementary material associated with this article can be found in the online version at doi:10.1016/j.adro.2021.100757.
