# Supplementary material for: A Beam-Specific Optimization Target Volume for Stereotactic Proton Pencil Beam Scanning Therapy for Locally Advanced Pancreatic Cancer
Source: Adv Radiat Oncol. 2021 Jul 29;6(6):100757. doi: 10.1016/j.adro.2021.100757 (PMC8463829; doi:10.1016/j.adro.2021.100757)
Supplement: Supplementary file 1 [file mmc1.docx]

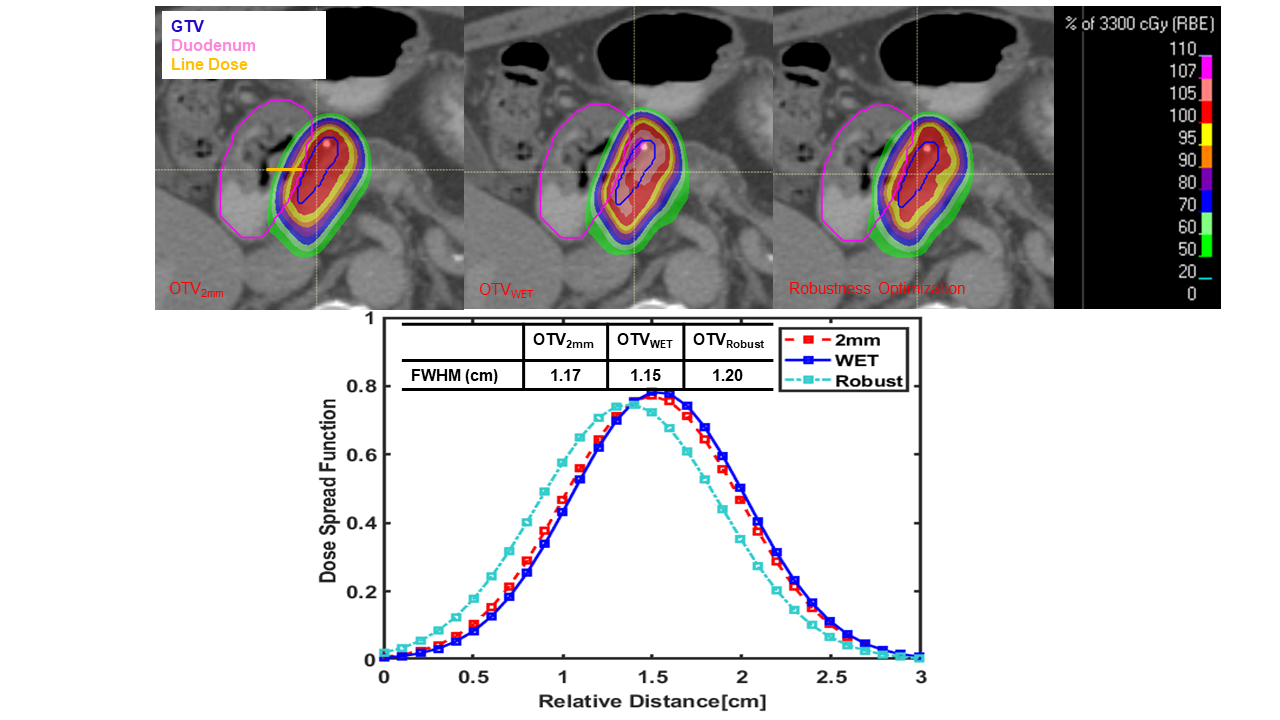


Figure A1. The DSF profiles from a typical patient case for all plans in the top row OTV_2mm_, OTV_WET,_ and RO from left to right. The line dose is indicated in solid yellow lines


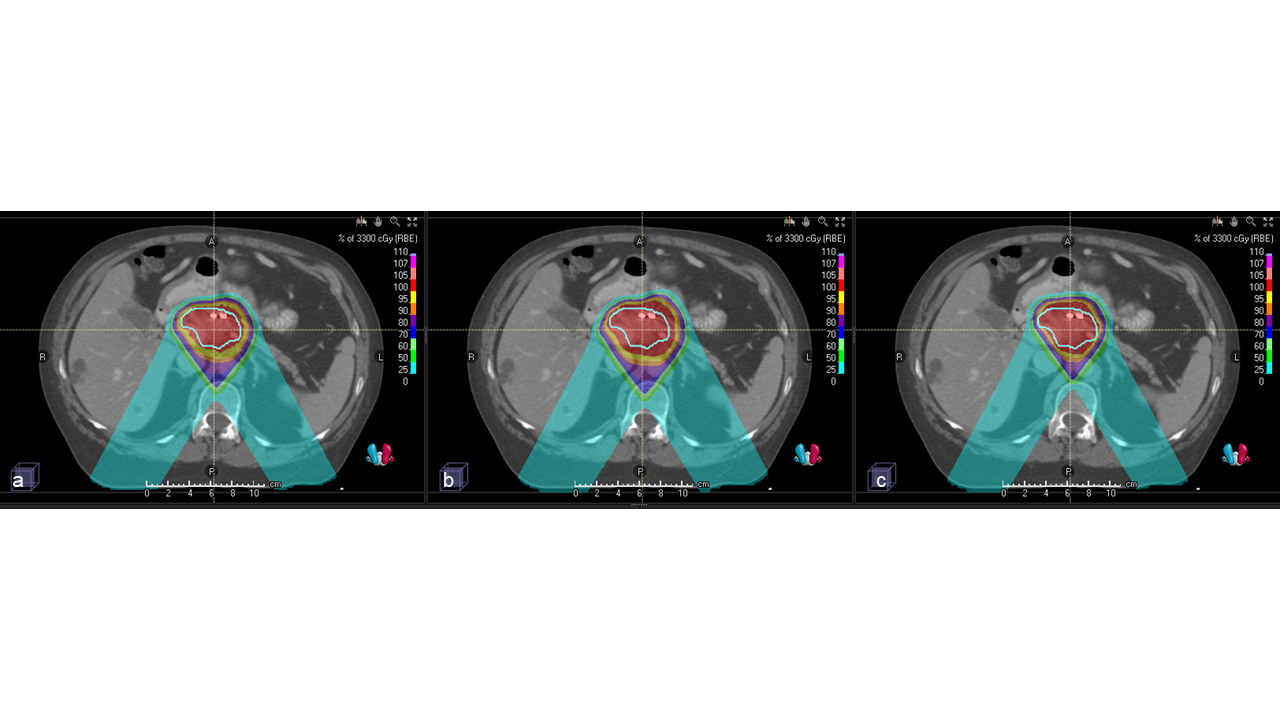


Figure A2. Dose distribution comparison for OTV_2mm_ (a) OTV_WET_ (b) and robustness optimization (c). The thick cyan line represents the boundary of GTV
