# Supplementary material for: A Beam-Specific Optimization Target Volume for Stereotactic Proton Pencil Beam Scanning Therapy for Locally Advanced Pancreatic Cancer
Source: Adv Radiat Oncol. 2021 Jul 29;6(6):100757. doi: 10.1016/j.adro.2021.100757 (PMC8463829; doi:10.1016/j.adro.2021.100757)
Supplement: Supplementary file 2 [file mmc2.docx]

|  | DVH metric | OTV_2mm_ | OTV_WET_ | OTV_5mm_ | Robustness  Optimization (RO) | p-values | | |
| --- | --- | --- | --- | --- | --- | --- | --- | --- |
| Scenarios to compute |  | 1 | 1 | 1 | 21 | OTV_2mm_ vs OTV_WET_ | OTV_2mm_ vs  RO | RO vs  OTV_WET_ |
| GTV | D98 [cGy] | 3299.5 | 3290.5 | 3271 | 3304.5 | 0.5006 | 0.0614 | <0.001 |
|  | D_mean_ [cGy] | 3319.8 | 3315.1 | 3348 | 3330.8 | 0.5588 | 0.2197 | 0.2557 |
|  | D_max_[cGy] | 3409.5 | 3399.5 | 3489 | 3483.8 | 0.7473 | 0.0648 | 0.0966 |
| Duodenum | V_33Gy(RBE)_ [cc] | 0.07 | 0.26 | 0.415 | 0.33 | <0.001 | 0.0342 | 0.4059 |
|  | V_20Gy(RBE)_ [cc] | 6.26 | 6.87 | 7.821 | 7.11 | <0.001 | 0.0291 | 0.5977 |
|  | V_15Gy(RBE)_ [cc] | 11.2 | 12 | 18.64 | 11.8 | <0.001 | 0.0311 | 0.6550 |
| Stomach | V_33Gy(RBE)_ [cc] | 0.12 | 0.14 | 0.077 | 0.19 | <0.001 | <0.001 | <0.001 |
|  | V_20Gy(RBE)_ [cc] | 2.51 | 3.05 | 2.502 | 2.93 | 0.3731 | 0.1264 | 0.9379 |
|  | V_15Gy(RBE)_ [cc] | 3.69 | 4.71 | 4.222 | 4.22 | 0.1232 | 0.1278 | 0.9328 |
| Small Bowel | V_33Gy(RBE)_ [cc] | 0.06 | 0.24 | 0.170 | 0.21 | <0.001 | <0.001 | 0.2504 |
|  | V_20Gy(RBE)_ [cc] | 2.23 | 3.28 | 3.701 | 2.81 | <0.001 | 0.1267 | 0.3831 |
|  | V_15Gy(RBE)_ [cc] | 3.43 | 4.85 | 5.03 | 4.31 | <0.001 | 0.1185 | 0.3174 |

Table A1 Summary of plan qualities (mean values if not specified) comparison for three optimization schemes of SBPT

|  | Band Width [%] | OTV_2mm_ | OTV_WET_ | Robustness  Optimization (RO) | p-values | | |
| --- | --- | --- | --- | --- | --- | --- | --- |
|  | | | | | OTV_2mm_ vs OTV_WET_ | OTV_2mm_ vs  RO | RO vs  OTV_WET_ |
| Duodenum | V_33_ [cc] | 0.44 | 0.17 | 0.37 | 0.7829 | 0.9617 | 0.0596 |
|  | V_20_ [cc] | 2.70 | 1.99 | 1.85 | 0.9062 | <0.0001 | 0.1124 |
|  | V_15_ [cc] | 3.18 | 2.31 | 2.38 | 0.7170 | 0.4434 | 0.6889 |

Table A2 Band width analysis on critical structure, duodenum

Table A3 Dose fall-off characteristic of all patients of the study

|  | OTV_2mm_ | OTV_WET_ | Robustness  Optimization (RO) |
| --- | --- | --- | --- |
| FWHM [cm] | 1.29 | 1.35 | 1.42 |
| FW20M [cm] | 1.97 | 2.07 | 2.16 |
